# Understanding the Toxicity Profile of Approved ADCs

**DOI:** 10.3390/pharmaceutics17020258

**Published:** 2025-02-14

**Authors:** Pablo Ballestín, Alfonso López de Sá, Cristina Díaz-Tejeiro, Lucía Paniagua-Herranz, Adrián Sanvicente, Igor López-Cade, Pedro Pérez-Segura, Carlos Alonso-Moreno, Cristina Nieto-Jiménez, Alberto Ocaña

**Affiliations:** 1 Experimental Therapeutics Unit, Departamento Oncología Médica, Instituto de Investigación Sanitaria San Carlos (IdISSC), Hospital Clínico San Carlos (HCSC), 28040 Madrid, Spain; 2 Unidad nanoDrug, Centro de Innovación en Química Avanzada (ORFEO-CINQA), Departamento de Química Inorgánica, Orgánica y Bioquímica, Facultad de Farmacia, Universidad de Castilla-La Mancha, 02071 Albacete, Spain; 3 Centro de Investigación Biomédica en Red en Oncología (CIBERONC), 28029 Madrid, Spain

**Keywords:** antibody–drug conjugate, antibody, toxicity, payload

## Abstract

**Background:** Antibody–drug conjugates (ADCs) represent a novel therapeutic class that combines an antibody against a tumor-associated antigen (TAA), a payload, and a linker that binds these two components. Serious adverse events (SAEs), particularly those of grade 3 (G3) or higher, frequently contribute to the abandonment of ADCs during clinical development. **Methods:** In this study, we analyzed the toxicity profiles of all approved ADCs, aiming to uncover correlations between their safety profiles and the specific characteristics of their components. **Results:** In our analysis, dose reductions, dose delays, treatment discontinuations, and ≥G3 toxicities were not significantly different across payload types. Similarly, no association was found between the payload mechanism of action and ≥G3 toxicities, including anemia, neutropenia, febrile neutropenia, thrombocytopenia, and diarrhea. By exploring the specific toxicities of ADCs observed by organ, we identified that most were related to the payload mechanism of action, like the ≥G3 diarrhea observed in 10% of patients treated with sacituzumab govitecan (the payload SN-38 is the active metabolite of irinotecan), and very few were related to the presence of the TAA in normal tissue (presence of Nectin-4 in skin and ≥G3 rash toxicity in 14% of patients treated with enfortumab vedotin). In line with this, no major differences in ≥G3 toxicities were identified in studies with different levels of the TAA (trastuzumab deruxtecan in Destiny Breast Studies with different HER2 expression levels). **Conclusions:** Our analysis reveals that most ADC toxicities are driven by the payload’s effects on non-transformed tissues; however, a detailed analysis of each ADC component should be taken into consideration.

## 1. Introduction

The principal reason for the failure of novel therapeutic agents in cancer is a lack of efficacy, followed by an inadequate safety profile [1]. A significant factor contributing to this inefficacy is the target itself: it may not play a critical role in human oncogenesis, or, if it does, the therapeutic agent may fail to engage with the target effectively [2]. A deeper understanding of oncogenic processes and the identification of synthetic lethality interactions can significantly enhance the selection of viable therapeutic targets [3]. For instance, agents targeting synthetic lethality interactions, such as PRMT5 inhibitors in tumors with MTAP deletions, are currently in early-stage clinical development, showing promising activity and favorable safety profiles [4,5]. Furthermore, advanced computational tools powered by artificial intelligence are being developed to enhance the design of chemical entities and innovative biologics [6].

Another major reason for the high attrition rate of new compounds is their safety profile. Adverse side effects often result from on- or off-target activity in non-tumoral tissues and the compound’s ability to access these tissues [7]. Addressing this issue requires improved patient selection strategies and efforts to optimize the therapeutic index, thereby reducing side effects and enhancing the success of new agents [7].

Antibody–drug conjugates (ADCs) represent a sophisticated therapeutic class that combines three key components: an antibody specifically designed to target a tumor-associated antigen (TAA); a payload, typically a highly potent chemical entity such as chemotherapy or a toxin; and a linker that binds these two components together [8,9]. This innovative approach has demonstrated clinical efficacy across multiple indications, with over 14 ADCs approved in recent years [8,10]. Despite this success, ADC development has encountered challenges due to toxicity, which has led to the discontinuation of some promising agents in clinical trials. The toxicity of ADCs can arise from any of their components: the antibody, the payload (determined by the type and the drug-to-antibody ratio [DAR]), or the linker [9,11].

Serious adverse events (SAEs), particularly those of grade 3 or higher, frequently contribute to the abandonment of ADCs during clinical development [12]. Even for approved ADCs, adverse events can limit their clinical use in specific subgroups of patients who exhibit heightened susceptibility to toxicity [12]. This article examines the toxicity profiles of all approved ADCs, aiming to uncover correlations between their safety profiles and the specific characteristics of their components. By doing so, we aim to offer valuable insights that could guide the development of safer and more effective ADCs.

## 2. Materials and Methods

### 2.1. Data Extraction and Analysis

We conducted a systematic search for FDA-approved antibody–drug conjugates (ADCs) using the FDA’s public database “Novel Drug Approvals”, which is updated annually (last accessed May 2024). From this database, we identified and selected the Phase 2 and Phase 3 clinical trials that supported the approvals of these ADCs.

For ADCs approved for multiple indications, we included all relevant clinical trials contributing to their approvals. To minimize bias related to toxicities caused by non-ADC drugs, we excluded later-phase clinical trials that tested approved ADCs in combination with other therapeutic agents.

### 2.2. Transcriptomic Evaluation of TAA

RNA sequencing expression data to evaluate the transcriptomic levels in tumors of the specific TAA were obtained from the TCGA and GTEx (Genotype-Tissue Expression) databases, using the bioinformatics tool ‘Gene Expression Profiling Interactive Analysis’ (http://gepia2.cancer-pku.cn/#index, accessed 1 November 2024).

### 2.3. Statistical Analysis

To compare the median drug–antibody ratio (DAR) between the anti-tubulin and DNA-damaging payload groups, we conducted a Wilcoxon rank sum test with continuity correction. The test was performed using RStudio statistical software (2024.12.0 version). A *p*-value of less than 0.05 was considered statistically significant.

## 3. Results

### 3.1. Dose Reduction, Dose Delay and Treatment Discontinuation

We explored whether there were any associations between the payload mechanism of action (anti-tubulin or DNA-damaging) and the frequency of any AE ≥ grade 3 (G3), AE dose reduction, AE dose delay/interruption, or AE withdrawal/discontinuation. No significant correlations were found between the payload type and any AE ≥ G3 (W = 1, *p* = 0.267), AE dose reduction (W = 1, *p* = 0.267), or AE dose delay/interruption (W = 1, *p* = 0.400). Greater variability was observed for AE withdrawal/discontinuation (W = 4, *p* = 1.000), though this was not statistically significant (Table 1 and Supplementary Table S1). In a similar manner, in hematologic tumors, no significant correlation was observed between the payload type and any AE ≥ G3 (W = 0, *p* = 0.100). Similarly, no associations were found with AE dose reduction (W = 5, *p* = 0.374), AE dose delay/interruption (W = 3, *p* = 0.667), or AE withdrawal/discontinuation (W = 0, *p* = 0.200) (Table 1 and Supplementary Table S2).

In summary, no statistically significant associations were identified between the payload mechanism of action and the analyzed safety parameters in either solid or hematologic tumors. However, a near-significant trend was observed for DNA-damaging payloads with any AE ≥ G3 in hematologic malignancies. Table 1 provides information about the frequency of the described safety data. As all the ADCs used cleavable linkers, except for belantamab mafodotin and trastuzumab emtansine, which used non-cleavable linkers, no statistical associations could be performed comparing the linker type.

### 3.2. Grade 3 Toxicity Type and Payload/Linker 

In solid tumors, no statistically significant association was found between the payload mechanism of action and ≥G3 toxicities, including anemia (W = 0, *p* = 0.100), neutropenia (W = 0, *p* = 0.133), febrile neutropenia (W = 0, *p* = 0.667), thrombocytopenia (W = 3, *p* = 1.000), and diarrhea (W = 2, *p* = 0.533). These results suggest that the mechanism of action of the payload does not significantly impact the incidence of these specific G3 toxicities in solid tumors.

In hematological malignancies, no statistically significant association was observed between the payload mechanism of action and any of the studied G3 toxicities, including anemia (W = 4, *p* = 1.000), neutropenia (W = 3, *p* = 0.700), febrile neutropenia (W = 4, *p* = 0.800), thrombocytopenia (W = 2, *p* = 1.000), and diarrhea (W = 5, *p* = 0.400). These findings indicate no significant impact of the payload mechanism on the occurrence of these grade 3 toxicities in hematological malignancies. 

### 3.3. Grade 3 Toxicity by Organ

Next, we explored in detail the specific toxicities of ADCs observed by organ. We focused on those presented in more than 5% of the population. In solid tumors, hematological toxicities and particularly ≥G3 neutropenia were observed for enfortumab vedotin, sacituzumab govitecan, and trastuzumab deruxtecan (Table 2). The toxicities in the hematological studies are described in Table 3. Trastuzumab deruxtecan produced more than 5% thrombocytopenia and anemia, and sacituzumab govitecan produced more than 5% anemia and febrile neutropenia (Table 2). For GI toxicities, sacituzumab govitecan produced 10% ≥G3 diarrhea, and trastuzumab deruxtecan produced around 5% nausea. For other AEs, fatigue was observed with trastuzumab deruxtecan and enfortumab vedotin in more than 6% of the treated patients. Corneal disorders were specifically observed for mirvetuximab soravtansine in 9% of the patients, and rash was observed for enfortumab vedotin in 14% of the patients (Supplementary Table S3).

We next aimed to explore whether this type of toxicity could be related to the payload, the target, or a combination of both depending on the agent. To do so, we analyzed data from publicly available transcriptomic studies as detailed in Section 2. When genomic datasets were examined, Nectin-4 was found to be highly expressed in the skin (expression in normal tissue of 107.35 TPM compared with other tissues) (Table 4). This finding could explain the presence of ≥G3 rash toxicity in 14% of the patients treated with enfortumab vedotin. However, the corneal disorders caused by mirvetuximab soravtansine do not seem to be related to FRα expression, due to its absence throughout the entire cornea. For sacituzumab govitecan, which produced ≥G3 diarrhea in 10% of patients, we observed that the expression of TROP2 in the colon was not highly present when compared with other tissues such as the esophageal or head and neck epithelium. This suggests that the observed toxicity could not be target-related. Indeed, this side effect is a common characteristic of irinotecan and SN-38, the payload of sacituzumab govitecan, which is the active metabolite of this chemotherapy agent [13].

In hematologic malignancies, ≥G3 neutropenia was observed in >20% of the patients for all ADCs except for belantamab mafodotin. Gemtuzumab ozogamizin caused ≥G3 thrombocytopenia in 100% of the patients and anemia in 86% of the population. Belantamab mafodotin against BCMA produced corneal disorders in 27% of the population. As can be said for mirvetuximab soravtansine, its target is not expressed in the eye [14]. Gemtuzumab ozogamizin caused ≥G3 toxicity in >10% of the patients, presenting as fatigue, bleeding, and an ALT/AST increase (Table 2) [15]. Of note, CD33 and BCMA are specifically present in acute myeloid leukemia and in plasma cells and some B cells [16], respectively, so the payload mechanism of action could produce an ALT/AST increase or corneal toxicity.

Of note, G3 corneal disorders were only observed for mirvetuximab soravtansine and belantamab mafodotin. Both of these ADCs share a payload with the same mechanism of action (anti-tubulin agents DM4 and MMAF, respectively) and a cleavable versus non-cleavable linker.

### 3.4. The Toxicity Profile of Trastuzumab Deruxtecan Does Not Depend on the Target Expression

Next, we sought to determine whether the toxicity profile of trastuzumab deruxtecan, the only ADC approved for breast cancer in both high and low HER2 expression settings, differed between these groups, and whether these differences could be attributed to target expression levels. To do so, we extracted the data from three different studies, Destiny Breast 04, 03, and 06. As can be seen in Table 5, no major differences in ≥G3 toxicities were identified. Only thrombocytopenia was not observed in low or ultralow patients. These data support our previous observations indicating that the toxicity profile mainly depends on the payload and does not relate to target presence.

## 4. Discussion

### 4.1. Evaluation of ADC Toxicity Profiles and Component Contributions

The primary aim of this study was to assess the toxicity profiles of FDA-approved ADCs and evaluate the potential contributions of their components to the observed adverse events. Starting with the payload, we analyzed the toxicities associated with different mechanisms of action. Notably, dose reductions, dose delays, treatment discontinuations, and ≥G3 toxicities were not significantly different across the payload types. However, we observed a trend approaching significance for DNA-damaging payloads in ≥G3 AEs for hematologic malignancies. This finding supports the notion that toxicity profiles and long-term tolerability are not solely dependent on the payload’s mechanism of action or the unique expression of the target antigen, as suggested by prior studies [11].

### 4.2. Linker Contributions to Toxicity

The analysis of linker types was limited because only two approved ADCs—belantamab mafodotin and trastuzumab emtansine—utilize non-cleavable linkers, precluding meaningful comparisons across groups. Linkers are considered critical for ADC safety because they influence payload release. Designing linkers requires a careful balance between chemical stability and the controlled release of the cytotoxic agent. The ADC must remain stable in the bloodstream to prevent premature release of the cytotoxic component, which could result in off-target toxicity. At the same time, the linker must allow for the effective release of the cytotoxic agent within the target cell after the ADC is internalized. The chemical composition of the linker, along with the conjugation method and site, plays a critical role in determining the DAR, as well as influencing the therapeutic index, pharmacokinetics, and pharmacodynamics of the ADC. In general terms, cleavable linkers may lead to faster payload release, potentially increasing off-target effects, while non-cleavable linkers generally improve ADC stability in circulation. In this context, recirculation of the ADCs can be higher for those with non-cleavable linkers. Additionally, ADCs with homogeneous drug-to-antibody ratios (DARs) exhibit reduced heterogeneity in blood levels, which may lower the risk of extra-exposure to the payload [17]. In addition, the optimization of linkers could increase efficacy and safety. For instance, exo-linkers provide a two-step activation mechanism, where the first cleavage event exposes a secondary recognition site, ensuring greater stability in circulation and precise drug release in the tumor microenvironment [18]. Tandem-cleavable linkers, on the other hand, incorporate multiple cleavage mechanisms (e.g., protease-sensitive + redox-responsive) to enhance payload release efficiency within tumors while minimizing systemic toxicity, offering a promising strategy for next-generation ADCs [19].

### 4.3. Target-Specific vs. Payload-Driven Toxicities

When grade ≥3 toxicities were analyzed, no significant differences were observed based on the payload’s mechanism of action. The transcriptomic data also did not indicate that these toxicities were confined to tissues expressing the tumor-associated antigen, with one notable exception: Nectin-4, which is highly expressed in skin tissues. The ADC targeting this antigen, enfortumab vedotin, caused grade ≥3 rash in 14% of patients. Beyond this example, tissue-specific toxicities appeared largely independent of antigen expression. For instance, gemtuzumab ozogamicin, which targets CD33 in acute myeloid leukemia, frequently causes ALT/AST elevations, despite CD33 being absent in hepatic tissue [20]. In this case, the toxicity is primarily driven by the payload; however, other factors must also be considered. We believe that calicheamicin, the payload, is the main contributor to liver enzyme elevations due to its role in inducing sinusoidal endothelial injury and hepatocyte apoptosis. Additionally, the hydrazone linker may exacerbate toxicity by enabling premature payload release, leading to increased systemic and hepatic exposure. However, we acknowledge that hematologic toxicities and systemic toxicities (nausea and febrile neutropenia) cannot be evaluated by the specific expression of the antigen target in a specific tissue. 

A comparison of the toxicity profile of trastuzumab deruxtecan across studies with different HER2 expression levels revealed no major differences. An exception was the absence of thrombocytopenia in the DESTINY-Breast06 study, which enrolled patients with low HER2 expression [21,22,23]. For hematologic malignancies, such as ADCs targeting CD33, high antigen expression in both leukemic and normal hematopoietic progenitor cells likely explains the trend toward increased grade ≥3 AEs observed with DNA-damaging payloads [24]. 

### 4.4. Payload-Specific Toxicities

Our analysis suggests that most ADC-related toxicities are attributable to the payload. For example, sacituzumab govitecan, which uses the SN-38 payload, often causes diarrhea, consistent with SN-38’s activity as the active metabolite of irinotecan [25]. Specific attention should also be given to corneal toxicity, which is observed with several ADCs:

- Mirvetuximab soravtansine (DM4 payload): corneal toxicity occurred in 9% of patients.
- Belantamab mafodotin (MMAF payload): corneal toxicity was observed in 27% of patients [26,27].
- Tisotumab vedotin (MMAE payload): corneal toxicity occurred in 2% of patients [28].
- Trastuzumab duocarmazine (a DNA-damaging payload): corneal toxicity was also reported [29].

Although this suggests that corneal toxicity may be linked to anti-tubulin payloads, the presence of this toxicity across multiple payload mechanisms highlights the complexity of its pathophysiology. It remains unclear whether the higher turnover of epithelial corneal cells predisposes them to payload uptake [30,31]. 

We acknowledge that our analysis did not reveal statistically significant differences when evaluating Grade >3 toxicities. However, this was a pooled analysis of high-grade toxicities, and lower-grade side effects were not considered. It is well established that DNA-damaging ADCs (e.g., topoisomerase inhibitors, calicheamicins) are associated with hematotoxicity and hepatotoxicity while exhibiting lower rates of peripheral neuropathy and ocular toxicity when compared to microtubule inhibitors such as auristatins and maytansinoids.

### 4.5. Mechanisms of ADC-Induced Toxicities

Several factors influencing ADC safety require further exploration:

1. Endocytosis and recycling: The role of endocytic processes in ADC safety is not fully understood. Preclinical studies suggest that Fcγ-receptor-mediated uptake by alveolar macrophages may result in the release of cytotoxic payloads into lung tissue, contributing to the interstitial lung disease observed with some ADCs [32]. This mechanism may also underlie the thrombocytopenia associated with trastuzumab emtansine [33]. However, it is important to mention that some ADCs have modifications of the Fc to avoid receptor interactions and, therefore, unexpected toxicity [34].
2. Micropinocytosis: This mechanism is a non-selective process, which means that it does not rely on specific receptors to internalize ADCs. This mechanism can play a role in the uptake of these agents by target cells, influencing their efficacy and, therefore, causing ocular toxicity [35].
3. Bystander effects: The physicochemical properties of payloads, such as hydrophobicity, can influence ADC stability and toxicity through bystander effects. Reducing the payload hydrophobicity has been shown to lower toxicity [36].

## 5. Conclusions

Our analysis revealed that most ADC toxicities are driven by the payload’s effects on non-transformed tissues, with only a minority of toxicities linked to antigen expression in normal tissues. For instance, ADCs targeting the same antigen but using different payloads exhibit distinct safety profiles, underscoring the critical role of the payload in determining toxicity.

On the other hand, we acknowledge that although the primary toxicity is driven by the payload, all components of the ADC individually or together can contribute to the overall safety profile. To enhance ADC safety, further research is needed to understand the contributions of payload mechanisms, linker stability, and target selection. This knowledge will be essential for designing ADCs that are both effective and safer, paving the way for broader clinical use in oncology.

## Figures and Tables

**Table 1:** Frequency of G3 or higher AEs and AEs leading to dose reduction, dose delay, or treatment discontinuation, according to target, payload, and DAR of FDA-approved ADCs, grouped by payload mechanism of action. Color code: green <5%; yellow 5–9.9%; orange 10–19.9%; red ≥20%.

Drug	Linker	Payload	Type of Payload	Target	DAR	Any AE ≥ G3 (%)	AE Dose Reduction (%)	AE Dose Delay/ Interruption (%)	AE Withdrawal/ Discontinuation (%)
Belantamab mafodotin	Non-cleavable linkers	MMAF	Anti-tubulin	BCMA	4	57.00	29.00	54.00	8.00
Brentuximab vedotin *◊	Cathepsin B-sensitive linker	MMAE		CD30	4	66.00	29.00	48.00	13.00
Enfortumab vedotin	Cathepsin B-sensitive linker	MMAE		NECTIN-4	3.8	51.40	32.40	51.00	13.50
Mirvetuximab soravtansine	Disulfide linker/glutathione-sensitive linker	Maytansinoid DM4		FRα	3.5	30.00	20.00	33.00	9.00
Polatuzumab vedotin ◊	Cathepsin B-sensitive linker	MMAE		CD79	3.5	60.70	9.20	NR	4.40
Tisotumab vedotin	Cathepsin B-sensitive linker	MMAE		Tissue Factor	4	28.00	22.00	24.00	12.00
Trastuzumab emtansine *	Non-cleavable linker	DM1		HER2	3.5	33.25	15.30	NR	11.90
Gemtuzumab ozogamizin *◊	Hydrazone linker/pH-sensitive linker	Calicheamicin	DNA-damaging	CD33	2–3	80.60	NR	NR	31.30
Inotuzumab ozogamizin	Disulfde linker/glutathione-sensitive linker	Calicheamicin		CD22	6	69.00	12.00	3.00	NR
Loncastuximab tesirine	Cathepsin B-sensitive linker	SG3199/PBD dimer		CD19	2.3	73.00	8.00	51.00	23.00
Sacituzumab govitecan *	Hydrazone linker/pH-sensitive linker	SN-38		Trop-2	7.6	59.50	34.00	55.50	6.00
Trastuzumab deruxtecan *	Cathepsin B-sensitive linker	DXd		HER2	8	49.40	25.90	33.65	13.95

* Data from ADCs with more than one clinical trial leading to FDA approvals are shown as the median values of the available data in published clinical trials. ◊ ADCs were administered in combination with other chemotherapy drugs or antibodies in at least one of the clinical trials included in the analysis. Abbreviations: DAR, drug–antibody ratio; AEs, adverse events; Any AE ≥ G3, frequency of patients with reported CTCAE grade 3 or higher adverse events in the antibody–drug conjugate arm safety population; AE dose reduction, frequency of patients with adverse events leading to dose reduction in the antibody–drug conjugate arm safety population; AE dose delay/interruption, frequency of patients with adverse events leading to dose delay or interruption in the antibody–drug conjugate arm safety population; AE withdrawal/discontinuation, frequency of patients with adverse events leading to treatment withdrawal or discontinuation in the antibody–drug conjugate arm safety population; NR, non-reported; BCMA, B-cell maturation antigen; FRα, folate receptor alpha.

**Table 2:** Frequency of G3 or higher hematologic, gastrointestinal, cardio-pulmonary, or other AEs, according to target, payload, and payload mechanism of action, of FDA-approved ADCs for solid tumors. Color code: green <5%; yellow 5–9.9%; orange 10–19.9%; red ≥20%.

Drug	Target	Pay-Load	Hematologic AEs ≥ G3 (%)				GI AEs ≥ G3 (%)			C-P AEs ≥ G3 (%)			Other AEs ≥ G3 (%)							
			A	N	FN	TC	D	Na	V	ILD	DEF	LVD	PN	F	R	CD	B	ASTi	ALTi	BRi
**Anti-tubulin Payload**																				
Enfortumab vedotin	NECTIN-4	MMAE	2.70	10.80	0.70	NR	3.40	1.00	NR	NR	NR	NR	5.10	6.40	14.50	0.00	NR	NR	NR	NR
Mirvetuximab soravtansine	FRα	DM4	1.00	2.00	NR	2.00	2.00	0.00	0.00	NR	NR	NR	0.00	0.90	NR	9.00	NR	NR	NR	NR
Tisotumab vedotin	Tissue Factor	MMAE	1.00	3.00	NR	1.00	1.00	0.00	2.00	NR	NR	NR	7.00	2.00	0.00%	2.00	2.00	NR	NR	NR
Trastuzumab emtansine *	HER2	DM1	1.90	1.60	NR	9.30	1.20	0.65	0.65	NR	0.60	0.15	1.40	1.75	NR	NR	1.40	2.40	1.65	NR
**DNA-damaging payload**																				
Sacituzumab govitecan *	Trop-2	SN-38	8.00	51.00	6.00	1.20	10.00	2.70	1.00	0.00	NR	NR	0.00	4.00	0.20	NR	NR	1.50	0.50	NR
Trastuzumab deruxtecan *	HER2	DXd	9.05	19.05	4.80	7.00	1.55	5.80	1.45	2.25	1.50	0.00	NR	7.25	NR	NR	NR	0.80	1.60	NR

* Data from ADCs with more than one clinical trial leading to FDA approvals are shown as the median values of the available data in published clinical trials. Abbreviations: AEs, adverse events; A, anemia; N, neutropenia; FN, febrile neutropenia; TC, thrombocytopenia; GI, gastrointestinal; C-P, cardio-pulmonary; D, diarrhea; Na, nausea; V, vomiting; ILD, interstitial lung disease or (preferred term) pneumonitis; DEF, decreased ejection fraction from baseline (CTCAE v5.0 G3 is defined by a ≥20% drop from baseline or resting EF of 39–20%); LVD, left ventricular dysfunction, or (preferred terms) cardiac failure and cardiac events, according to the reporting methodology of each clinical trial; PN, peripheral neuropathy; F, fatigue; R, rash; CD, corneal disorders; B, bleeding; ASTi, aspartate aminotransferase increase; ALTi; alanine aminotransferase increase; BRi, bilirubin increase; NR, non-reported; FRα, folate receptor alpha.

**Table 3:** Frequency of G3 or higher hematologic, gastrointestinal, cardio-pulmonary, and other AEs, according to target, payload, and payload mechanism of action, of FDA-approved ADCs for hematologic malignancies. Color code: green <5%; yellow 5–9.9%; orange 10–19.9%; red ≥20%.

Drug	Target	Payload	Hematologic AEs ≥ G3 (%)				GI AEs ≥ G3 (%)			C-P AEs ≥ G3 (%)			Other AEs ≥ G3 (%)							
			A	N	FN	TC	D	Na	V	ILD	DEF	LVD	PN	F	R	CD	B	ASTi	ALTi	BRi
**Anti-tubulin Payload**																				
Belantamab mafodotin	BCMA	MMAF	21.00	11.00	NR	22.00	1.00	0.00	2.00	NR	NR	NR	NR	2.00	NR	27.00	NR	2.00	NR	NR
Brentuximab vedotin *◊	CD30	MMAE	8.00	44.50	18.50	NR	3.00	2.00	2.00	0.75	NR	NR	9.09	3.00	2.00	NR	NR	NR	3.00	NR
Polatuzumab vedotin ◊	CD79	MMAE	12.00	28.30	13.80	NR	3.90	1.10	1.10	NR	NR	NR	1.60	0.90	NR	NR	NR	NR	NR	NR
**DNA-damaging payload**																				
Gemtuzumab ozogamizin *◊	CD33	Calicheamicin	86.20	96.10	18.00	100	NR	NR	NR	NR	NR	7.15	NR	11.70	NR	NR	17.75	14.00	10.90	7.10
Inotuzumab ozogamizin	CD22	Calicheamicin	11.00	34.00	14.00	20.00	0.00	3.00	0.00	NR	NR	NR	NR	1.00	0.00	NR	NR	1.00	1.00	3.00
Loncastuximab tesirine	CD19	SG3199/PBD dimer	10.00	26.00	3.00	18.00	2.00	0.00	0.00	1.00	NR	NR	1.00	1.00	1.00	NR	NR	1.00	3.00	1.00

* Data from ADCs with more than one clinical trial leading to FDA approvals are shown as the median values of the available data in published clinical trials. ◊ ADCs were administered in combination with other chemotherapy drugs or antibodies in at least one of the clinical trials included in the analysis. Abbreviations: AEs, adverse events; A, anemia; N, neutropenia; FN, febrile neutropenia; TC, thrombocytopenia; GI, gastrointestinal; C-P, cardio-pulmonary; D, diarrhea; Na, nausea; V, vomiting; ILD, interstitial lung disease or (preferred term) pneumonitis; DEF, decreased ejection fraction from baseline (CTCAE v5.0 G3 is defined by a ≥20% drop from baseline or resting EF of 39–20%); LVD, left ventricular dysfunction, or (preferred terms) cardiac failure and cardiac events, according to the reporting methodology of each clinical trial; PN, peripheral neuropathy; F, fatigue; R, rash; CD, corneal disorders; B, bleeding; ASTi, aspartate aminotransferase increase; ALTi; alanine aminotransferase increase; BRi, bilirubin increase; NR, non-reported; BCMA, B-cell maturation antigen.

**Table 4:** Target expression of ADCs in normal tissue (TPM).

Drug	Target	Expression in Normal Tissue (TPM)				
		Skin	Head and Neck	Esophagus	Stomach	Colon
Enfortumab vedotin	NECTIN-4	107.35	51.15	86.36	2.58	0.31
Tisotumab vedotin	Tissue factor	52.21	53.66	32.35	26.67	26.89
Trastuzumab emtansine *	HER-2	88.61	70.96	88.52	26.77	33.56
Trastuzumab deruxtecan *	HER-2	88.61	70.96	88.52	26.77	33.56
Sacituzumab govitecan *	Trop-2	344.19	589.62	725.26	5.07	0.67
Mirvetuximab soravtansine	FRα	1.77	1.79	0.28	8.55	0.18

* Data from ADCs with more than one clinical trial leading to FDA approvals are shown as the median values of the available data in published clinical trials. Blue means expression higher than 32 TPM. TPM, transcripts per million; AEs, adverse events; FRα, folate receptor alpha.

**Table 5:** Toxicity profile of trastuzumab deruxtecan in clinical studies selecting patients with high expression (Destiny Breast 03) versus low expression (Destiny Breast 04 and 06). Color code: green <5%; yellow 5–9.9%; orange 10–19.9%; red ≥20%.

Study	Patient Profile	HER2 Status	N (%)	A (%)	T (%)	Na (%)	D (%)	V (%)	P (%)	ALTi/ASTi (%)	As (%)
Destiny Breast 04	2nd-/3rd-line QT	HER2-negative (90% RH+/10% RH−)	13.7	8.1	5.1	4.6	1.1	1.3	2.1	3.2	7.5
		HER2 1+ (58%), HER2 2+/ISH− (42%)	NR	NR	NR	NR	NR	NR	NR	NR	NR
Destiny Breast 03	2nd line	HER2-positive (88–90% 3+ by IHQ, 9–11% 2+/ISH+)	19.1	5.8	7.0	6.6	0.4	1.6	0.8	1.6	5.1
Destiny Breast 06	1st-line QT	HR+/HER2− (HER2 low 82%, HER2 ultralow 18%)	20.7	5.8	NR	1.6	1.8	1.4	1.4	2.3	3.7

Abbreviations: A, anemia; N, neutropenia; T, thrombocytopenia; D, diarrhea; Na, nausea; V, vomiting; P, pneumonitis; ASTi, aspartate aminotransferase increase; ALTi; alanine aminotransferase increase; As, asthenia; HR, hormonal receptor; NR, non-reported.

## Data Availability

The original contributions presented in this study are included in the article/Supplementary Materials. Further inquiries can be directed to the corresponding author(s).

## Supplementary Materials

The following supporting information can be downloaded at: https://www.mdpi.com/article/10.3390/pharmaceutics17020258/s1, Table S1: Frequency of any G3 or higher AEs, and AEs leading to dose reduction, dose delay, and treatment discontinuation, according to target, payload, and DAR of FDA approved ADCs for the treatment of solid tumors, divided by payload mechanism of action; Table S2: Frequency of any G3 or higher AEs, and AEs leading to dose reduction, dose delay, and treatment discontinuation, according to target, payload, and DAR of FDA approved ADCs for hematologic malignancies, divided by payload mechanism of action; Table S3: Grade 3 toxicities of ADCs by organ.
