# Supplementary material for: Understanding the Toxicity Profile of Approved ADCs
Source: Pharmaceutics. 2025 Feb 14;17(2):258. doi: 10.3390/pharmaceutics17020258 (PMC11858992; doi:10.3390/pharmaceutics17020258)
Supplement: Supplementary file 1 [file pharmaceutics-17-00258-s001.zip › pharmaceutics-3447716-supplementary.pdf]

Supplementary Table S1. Frequency of any G3 or higher AEs, and AEs leading to dose reduction, dose delay, and treatment discontinuation, according to target, payload, and DAR of FDA approved ADCs for the treatment of **solid tumors**, divided by payload mechanism of action.

**Drug**	**Target**	**Payload**	**DAR**	**Any AE ≥G3**	**AEs Dose Reduction**	**AEs Dose Delay/Interruption**	**AEs Withdrawal/Discontinuation**
**Antitubulin payload**							
Enfortumab vedotin	NECTIN-4	MMAE	3.8	51.40%	32.40%	51.00%	13.50%
Mirvetuximab soravtamsine	FRα	Maytansinoid DM4	3.5	30.00%	20.00%	33.00%	9.00%
Tisotumab vedotin	Tissue Factor	MMAE	4	28.00%	22.00%	24.00%	12.00%
Trastuzumab emtansine*	HER2	DM1	3.5	33.25%	15.30%	NR	11.90%
**DNA damaging payload**							
Sacituzumab govitecan*	Trop-2	SN-38	7.6	59.50%	34.00%	55.50%	6.00%
Trastuzumab deruxtecan*	HER2	DXd	8	49.40%	25.90%	33.65%	13.95%

* Data from ADCs with more than one clinical trial leading to FDA approvals is shown as the median values of available data in published clinical trials.

DAR, drug antibody ratio; AEs, adverse events; Any AE ≥G3, frequency of patients with reported CTCAE grade 3 or higher adverse events in the antibody-drug conjugate arm safety population; AEs Dose Reduction, frequency of patients with adverse events leading to dose reduction in the antibody-drug conjugate arm safety population; AEs Dose Delay/Interruption, frequency of patients with adverse events leading to dose delay or interruption in the antibody-drug conjugate arm safety population; AEs Withdrawal/Discontinuation, frequency of patients with adverse events leading to treatment withdrawal or discontinuation in the antibody-drug conjugate arm safety population; NR, non-reported; FRα, folate receptor alpha.

Supplementary Table S2. Frequency of any G3 or higher AEs, and AEs leading to dose reduction, dose delay, and treatment discontinuation, according to target, payload, and DAR of FDA approved ADCs for **hematologic malignancies**, divided by payload mechanism of action.

Drug	Target	Payload	DAR	Any AE ≥G3	AEs Dose Reduction	AEs Dose Delay/Interruption	AEs Withdrawal/Discontinuation
**Antitubulin payload**							
Belantamab mafodotin	BCMA	MMAF	4	57.00%	29.00%	54.00%	8.00%
Brentuximab vedotin*◊	CD30	MMAE	4	66.00%	29.00%	48.00%	13.00%
Polatuzumab vedotin ◊	CD79	MMAE	3.5	60.70%	9.20%	NR	4.40%
**DNA damaging payload**							
Gemtuzumab ozogamizin*◊	CD33	Calicheamicin	2-3	80.60%	NR	NR	31.30%
Inotuzumab ozogamizin	CD22	Calicheamicin	6	69.00%	12.00%	3.00%	NR
Loncastuximab tesirine	CD19	SG3199/PBD dimer	2.3	73.00%	8.00%	51.00%	23.00%

* Data from ADCs with more than one clinical trial leading to FDA approvals is shown as the median values of available data in published clinical trials.

◊ ADCs were administered in combination with other chemotherapy drugs or antibodies in at least one of the clinical trials included in the analysis.

DAR, drug antibody ratio; AEs, adverse events; Any AE ≥G3, frequency of patients with reported CTCAE grade 3 or higher adverse events in the antibody-drug conjugate arm safety population; AEs Dose Reduction, frequency of patients with adverse events leading to dose reduction in the antibody-drug conjugate arm safety population; AEs Dose Delay/Interruption, frequency of patients with adverse events leading to dose delay or interruption in the antibody-drug conjugate arm safety population; AEs Withdrawal/Discontinuation, frequency of patients with adverse events leading to treatment withdrawal or discontinuation in the antibody-drug conjugate arm safety population; NR, non-reported; BCMA, B-cell maturation antigen.

Supplementary Table S3. Grade 3 toxicities of ADCs by organ.

Drug	Linker	Payload	AE ≥ G3								
			Neutropenia	Thrombocytopenia	Anemia	Febrile Neutropenia	Diarrhea	Nausea	Fatigue	Corneal disorders	Rash
Enfortumab vedotin	Cathepsin B-sensitive linker	MMAE	10.8						6.4		14.5
Mirvetuximab soravtansine	Disulfide linker/ glutathione sensitive linker	Maytansinoid DM4								9	
Sacituzumab govitecan*	Hydrazone linker/ pH-sensitive linker	SN-38	51		8	6	10				
Trastuzumab deruxtecan*	Cathepsin B-sensitive linker	DXd	19.05	7	9.05			5.8	7.25		

* Data from ADCs with more than one clinical trial leading to FDA approvals is shown as the median values of available data in published clinical trials.
